# An open-label expanded access program of afatinib in EGFR tyrosine kinase inhibitor-naïve patients with locally advanced or metastatic non-small cell lung cancer harboring *EGFR* mutations

**DOI:** 10.1186/s12885-021-08445-9

**Published:** 2021-07-12

**Authors:** Keunchil Park, Jin-Soo Kim, Joo-Hang Kim, Young-Chul Kim, Hoon-Gu Kim, Eun Kyung Cho, Jong-Youl Jin, Miyoung Kim, Angela Märten, Jin-Hyoung Kang

**Affiliations:** 1 Division of Hematology-Oncology, Department of Medicine Samsung Medical Center, Sungkyunkwan University School of Medicine, 81 Irwon-ro, Gangnam-gu, 06351 Seoul, South Korea; 2 Department of Internal Medicine, Seoul Metropolitan Government Seoul National University Boramae Medical Center, Seoul, South Korea; 3 CHA Bundang Medical Center, CHA University, Gyeonggi-do, Seongnam, South Korea; 4 Chonnam National University Medical School, CNU Hwasun Hospital, Gwangju, South Korea; 5 Department of Internal Medicine, Gyeongsang National University College of Medicine and Gyeongsang National University Changwon Hospital, Changwon, South Korea; 6 Gil Medical Center, Gachon University College of Medicine, Incheon, South Korea; 7 Bucheon St Mary’s Hospital, The Catholic University of Korea, Bucheon, South Korea; 8 Boehringer Ingelheim Korea Ltd, Seoul, South Korea; 9 Boehringer Ingelheim International GmbH, Ingelheim am Rhein, Germany; 10 Department of Internal Medicine, Seoul St. Mary’s Hospital, The Catholic University of Korea, Seoul, South Korea

**Keywords:** Afatinib, NSCLC, EGFR, Uncommon mutations, Brain metastases, Real world

## Abstract

**Background:**

Afatinib is approved globally for EGFR-TKI treatment-naïve patients with *EGFR* mutation-positive non-small cell lung cancer (NSCLC). In this Korean expanded access program, we evaluated its ‘real-world’ safety and efficacy.

**Methods:**

EGFR-TKI treatment-naïve patients with *EGFR* mutation-positive NSCLC received afatinib 40 mg/day until disease progression or other withdrawal criteria. Dose reductions were permitted for adverse events (AEs). The primary endpoint was the number of patients with AEs (CTCAE version 3.0). Other endpoints included progression-free survival (PFS), overall response rate (ORR), duration of response (DOR), and changes in investigator-assessed cancer-related symptoms.

**Results:**

Eighty-eight patients received afatinib, including 27 (31%) with brain metastases and 16 (18%) with uncommon *EGFR* mutations. Median PFS was 17.0 months (95% confidence interval [CI] 12.9–23.3 months). Grade 3 treatment-related AEs (TRAEs) were reported in 51 (58%) patients; the most common were diarrhea (22%) and rash/acne (20%). No grade > 3 TRAEs were reported. AEs leading to dose reduction occurred in 49 (56%) patients. Treatment discontinuation due to TRAEs occurred in 4 (5%) patients. ORR was 81% overall, 89% in patients with brain metastases, and 55% in patients with uncommon mutations (excluding T790M/exon 20 insertions). Median DOR was 15.1 months (95% CI 12.4–21.4 months). Cancer-related symptoms were improved/unchanged/worsened in 34–66%/36–66%/0–3% of patients over the first year.

**Conclusions:**

No unexpected safety signals for afatinib were observed. AEs were manageable; the treatment discontinuation rate was low. Afatinib showed encouraging efficacy in a broad patient population including those with brain metastases or tumors harboring uncommon *EGFR* mutations.

**Trials registration:**

ClinicalTrials.gov NCT01931306; 29/08/2013.

**Supplementary Information:**

The online version contains supplementary material available at 10.1186/s12885-021-08445-9.

## Background

In Korea, lung cancer is the most common cause of cancer-related death [1]. While non-small-cell lung cancer (NSCLC) is highly heterogeneous, mutations in the epidermal growth factor receptor (*EGFR*) gene, leading to aberrant EGFR signaling, occur in up to 50% of Asian patients [2, 3]. Tumors harboring *EGFR* mutations are highly sensitive to EGFR tyrosine kinase inhibitors (TKIs) [4–6]. Currently, five EGFR TKIs are approved for the treatment of *EGFR* mutation-positive NSCLC: the first-generation reversible EGFR TKIs, gefitinib and erlotinib; the second-generation irreversible ErbB family blockers, afatinib and dacomitinib, and the third-generation wild-type sparing irreversible EGFR TKI, osimertinib [4–6].

In randomized trials, first-line afatinib has demonstrated improved progression-free survival (PFS) compared with chemotherapy (LUX-Lung 3 and 6 [7, 8]) and gefitinib (LUX-Lung 7 [9]) in patients with *EGFR* mutation-positive NSCLC. Of note, in both LUX-Lung 3 and 6, prespecified analysis demonstrated that afatinib significantly improved overall survival (OS) versus chemotherapy in patients with tumors carrying a Del19 *EGFR* mutation [10]. In LUX-Lung 7, median OS was numerically longer with afatinib versus gefitinib, but did not achieve statistical significance [11]. Across clinical trials, afatinib was well tolerated [7–9]. Treatment-related adverse events (TRAEs) with afatinib were predominantly class-related gastrointestinal and cutaneous events. While certain TRAEs (e.g., diarrhea) were more common than generally observed with first- and third-generation TKIs, they were manageable with dose reductions, which did not compromise clinical activity [12, 13]. Consequently, treatment discontinuations due to TRAEs were rare (< 10%). Together, these observations support the use of afatinib for patients with *EGFR* mutation-positive NSCLC. In phase III trials, both dacomitinib (exploratory analysis) [14] and osimertinib [15] have demonstrated prolonged OS benefit versus first-generation EGFR TKIs. However, as the efficacy and safety of afatinib, dacomitinib, and osimertinib have never been directly compared in prospective trials, optimal first-line treatment choice remains an open question, particularly in Asian patients, as there was no evidence of OS benefit with osimertinib over erlotinib/gefitinib in this subgroup. Therefore, it is important to assess the efficacy and safety of EGFR TKIs in broader ‘real-world’ patient populations that are more representative of routine practice than clinical trials [16]. Here, we describe the results of an expanded access program performed in South Korea.

## Methods

### Study design

This was an open-label, single-arm, expanded access, phase IIIb trial conducted at eight centers in South Korea. The objectives were to evaluate the tolerability and activity of afatinib in EGFR TKI-naïve patients with *EGFR* mutation-positive NSCLC.

### Patients and treatment

Patients were ≥ 18 years old with locally advanced or metastatic *EGFR* mutation-positive NSCLC, were EGFR TKI-naïve (one previous line of chemotherapy was permitted), and had an Eastern Cooperative Oncology Group performance status (ECOG PS) of 0–2 and adequate organ function. Main exclusion criteria were hypersensitivity to afatinib, hormonal anticancer treatment or radiotherapy within 14 days and surgery within 4 weeks prior to study start, cardiovascular abnormalities, pre-existing interstitial lung disease (ILD), active chronic infections, previous/concomitant malignancies, and symptomatic brain metastases. Patients received oral afatinib 40 mg daily in 28-day cycles until disease progression, lack of tolerability, or other study withdrawal criteria. Treatment with afatinib was permitted beyond disease progression if deemed beneficial during assessment. Treatment interruptions and dose reductions were permitted for the management of TRAEs (see the Supporting methods section in the Additional file 1).

At each center, ethical approval was given by an Institutional Review Board or and Independent Ethics Committee. The study was performed in accordance with the Declaration of Helsinki, Good Clinical Practice, and local laws. Written informed consent was given by all patients.

### Endpoints, assessments and statistical considerations

The primary endpoint was the frequency of adverse events (AEs), coded using the Medical Dictionary for Regulatory Activities (MedDRA) version 21.1 and graded by the Common Terminology Criteria for Adverse Events (CTCAE) version 3. Treatment-emergent adverse events (TEAEs), TRAEs, and serious AEs (SAEs) were reported. MedDRA preferred terms for AEs of a similar nature were grouped; grouped terms included rash/acne, stomatitis, and renal insufficiency. Other endpoints included PFS, tumor response, and assessment of cancer-related symptoms (see the Supporting methods section in the Additional file 1). Cancer-related symptoms were recorded in the electronic case report form based on investigator judgment as either improved, unchanged, or ‘worsening of symptoms due to cancer’. Patients were categorized every 4 weeks and at the end-of-treatment (EOT) visit. *EGFR* mutations were detected according to the methodology at each participating site.

Analyses were performed on all patients who received at least one dose of afatinib (treatment set). Kaplan–Meier estimates and 95% confidence intervals (CIs) were calculated for time-to-event endpoints using Greenwood’s standard error estimate. Two-sided 95% CIs were calculated for the overall response rate (ORR) using the exact 95% Clopper-Pearson CI. Post hoc analyses of treatment outcomes were performed in patient subgroups based on age (< 65 years and ≥ 65 years), presence of brain metastases (yes and no), *EGFR* mutation status (common [Del19 only or L858R only] and uncommon), and dose interruptions within the first 6 months (yes and no).

## Results

### Patients and treatment exposure

**Fig. 1:**
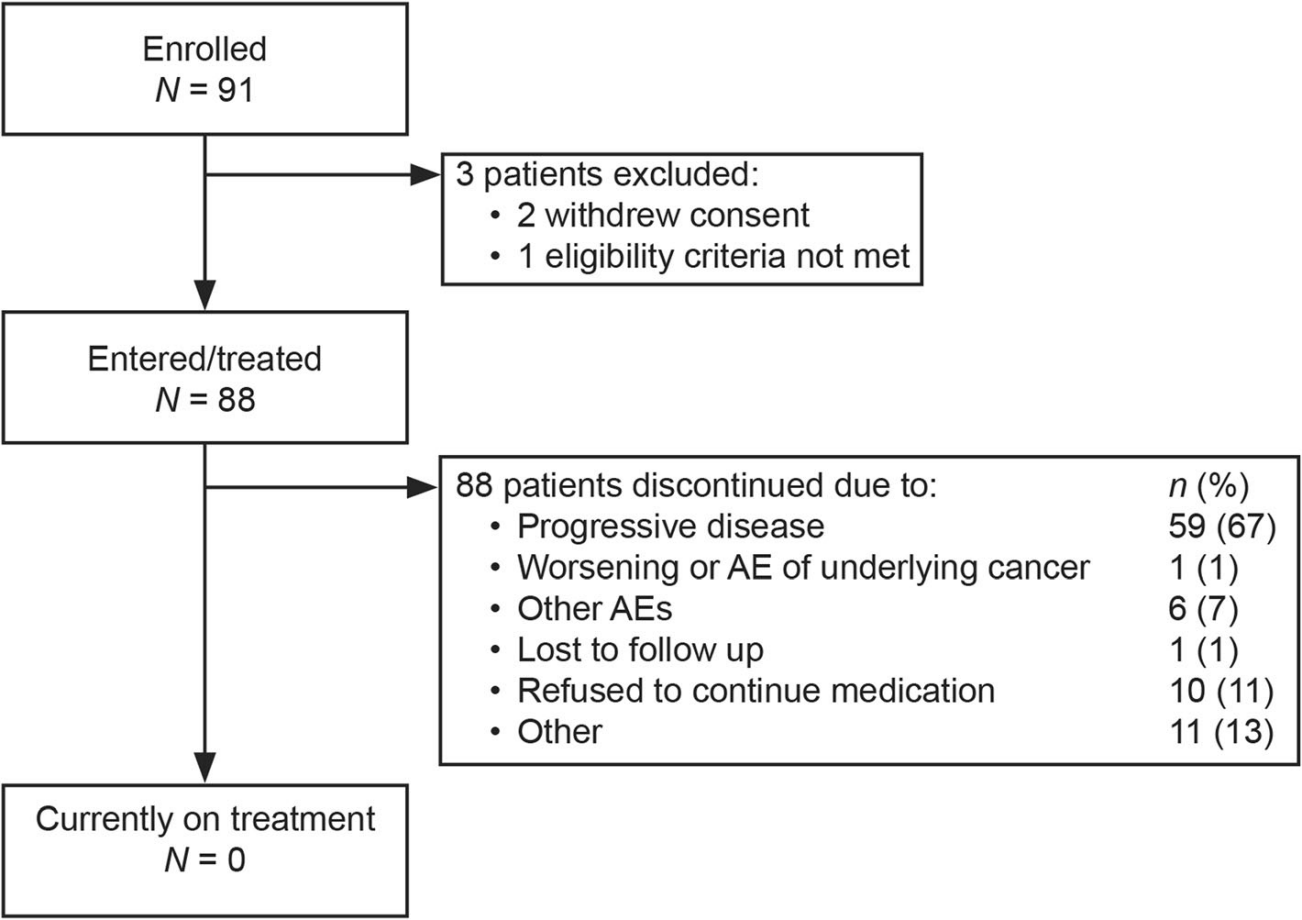
Patient disposition. (AE: adverse event)

Between August 28, 2013 and September 21, 2018, 91 patients were enrolled and 88 were treated with afatinib (Fig. 1). All patients discontinued treatment, primarily due to disease progression (67%). Median age was 61 years (range, 37–87). Seventy (80%) patients had stage IV disease, 27 (31%) had asymptomatic brain metastases, and 81 (98%) had an ECOG PS of 0 or 1 (Table 1). Fifty (57%) patients harbored an *EGFR* Del19 mutation; 16 (18%) patients had an uncommon *EGFR* mutation (Additional file 1: Table S1). Median treatment duration was 15.3 months (range, 0.3–56.1). Forty-nine (56%) patients had a dose reduction to 30 mg/day. Of these patients, 22 (25%) had a second dose reduction to 20 mg.

**Table 1:** Patient baseline and disease characteristics

Characteristic	Treatment Set *N* = 88
Age	
Median (range), years	61 (37–87)
< 65	53 (60)
≥ 65	35 (40)
≥ 75	5 (6)
Sex	
Female	46 (52)
Male	42 (48)
Smoking status	
Never	55 (63)
Ex-smoker	27 (31)
Current smoker	6 (7)
ECOG PS score	
0	15 (17)
1	71 (81)
2	2 (2)
Tumor histology	
Predominantly adenocarcinoma	87 (99)
Predominantly squamous	1 (1)
Clinical stage at initial diagnosis	
IA	7 (8)
IB	4 (5)
IIA	1 (1)
IIB	1 (1)
IIIA	2 (2)
IIIB	3 (3)
IV	70 (80)
Metastases	
Any	86 (98)
Brain	27 (31)
EGFR mutation categories	
Del19	51 (58)
L858R	27 (31)
Exon 20 insertions	3 (3)
L861Q	6 (7)
G719X	4 (5)
T790M	2 (2)
S768I	0
Other	2 (2)
EGFR mutation	
Common	72 (82)
Uncommon	16 (18)
Any previous systemic chemotherapies	30 (34)

*ECOG PS* Eastern Cooperative Oncology Group performance status, *EGFR* epidermal growth factor receptor

Data are *n* (%) unless otherwise stated

### Adverse events

All 88 treated patients had at least one TEAE (Table 2). Grade 3 TEAEs were reported in 55 (63%) patients (Additional file 1: Table S2). Grade 4 TEAEs were reported in two patients (one bradycardia and one cardiac arrest). TEAEs leading to dose-reductions were reported in 49 (56%) patients. The most prevalent reasons for dose reduction were diarrhea (23%), rash/acne (18%), and stomatitis (11%). TEAEs leading to death were reported in seven patients including two with neoplasm progression, two respiratory failure, and one each of dyspnea, hydrocephalus, and death (MedDRA preferred term). SAEs were reported in 37 (42%) patients; 17 (19%) patients had grade 3 SAEs and two (2%) had grade 4 SAEs (Table 2). Eight (9%) patients had treatment-related SAEs.

**Table 2:** Summary of TEAEs independent of relatedness

AE	Treatment Set (*N* = 88)
Any AE	88 (100)
Leading to dose reduction	49 (56)
Leading to discontinuation	8 (9)
SAE	37 (42)
Fatal	7 (8)
Immediately-life threatening	1 (1)
Disability incapacity	0
Requiring hospitalization	37 (42)
Prolonging hospitalization	3 (3)
Congenital anomaly	0
Other	2 (2)
Highest CTCAE grade	
Grade 1	2 (2)
Grade 2	22 (25)
Grade 3	55 (63)
Grade 4	2 (2)
Grade 5	7 (8)

*AE* adverse event, *CTCAE* Common Terminology Criteria for Adverse Events, *SAE* serious adverse event, *TEAE* treatment-emergent adverse events

Data shown are *n* (%)

TRAEs were reported in 87 (99%) patients; 51 (58%) experienced a grade 3 TRAE (Table 3). No grade 4 or 5 TRAEs were reported. The most common TRAEs were (any grade/grade 3) diarrhea (98%/22%), rash/acne (92%/20%), and stomatitis (76%/15%). Most TRAEs occurred soon after initiation of therapy and were managed with supportive measures and/or reduction of dose. The onset of treatment-related diarrhea and rash/acne occurred within 1 week of initiation in 70 and 40% of cases, respectively; supportive treatment was administered in 84 and 80% of cases, respectively. Four (5%) patients discontinued afatinib due to TRAEs: grade 3 decreased appetite, generalized edema, ILD, and paronychia, respectively. There were no treatment-related deaths.

**Table 3:** Treatment-related adverse events

AE	Treatment Set (*N* = 88)	
	Grade 3^b^	All Grades
Any TRAE	51 (58)	87 (99)
Diarrhea	19 (22)	86 (98)
Rash/acne^a^	18 (20)	81 (92)
Stomatitis^a^	13 (15)	67 (76)
Pruritus	2 (2)	40 (45)
Paronychia^a^	8 (9)	36 (41)
Nail disorder	3 (3)	30 (34)
Decreased appetite	5 (6)	20 (23)
Dry skin	0	18 (20)
Nasal inflammation	0	9 (10)
Palmar-plantar erythrodysesthesia syndrome	1 (1)	8 (9)
Nausea	1 (1)	7 (8)
Abdominal pain upper	0	5 (6)
Alopecia	0	5 (6)
Fatigue	2 (2)	5 (6)
Rhinorrhea	0	5 (6)
Scab	0	5 (6)
Skin hyperpigmentation	0	5 (6)

*AE* adverse event, *CTCAE* Common Terminology Criteria for Adverse Events, *MedDRA* Medical Dictionary for Regulatory Activities, *TRAE* treatment-related adverse event

Shown are *n* (%) TRAEs in > 5% of patients at any grade in the patient treatment set. TRAEs are shown by MedDRA version 21.1 preferred terms and highest grade according to CTCAE version 3. ^a^Category of specific grouped preferred terms as specified in the Patients and Methods; ^b^No CTCAE grade 4 or 5 TRAEs were reported

### Patient outcomes and subgroup analysis

**Fig. 2:**
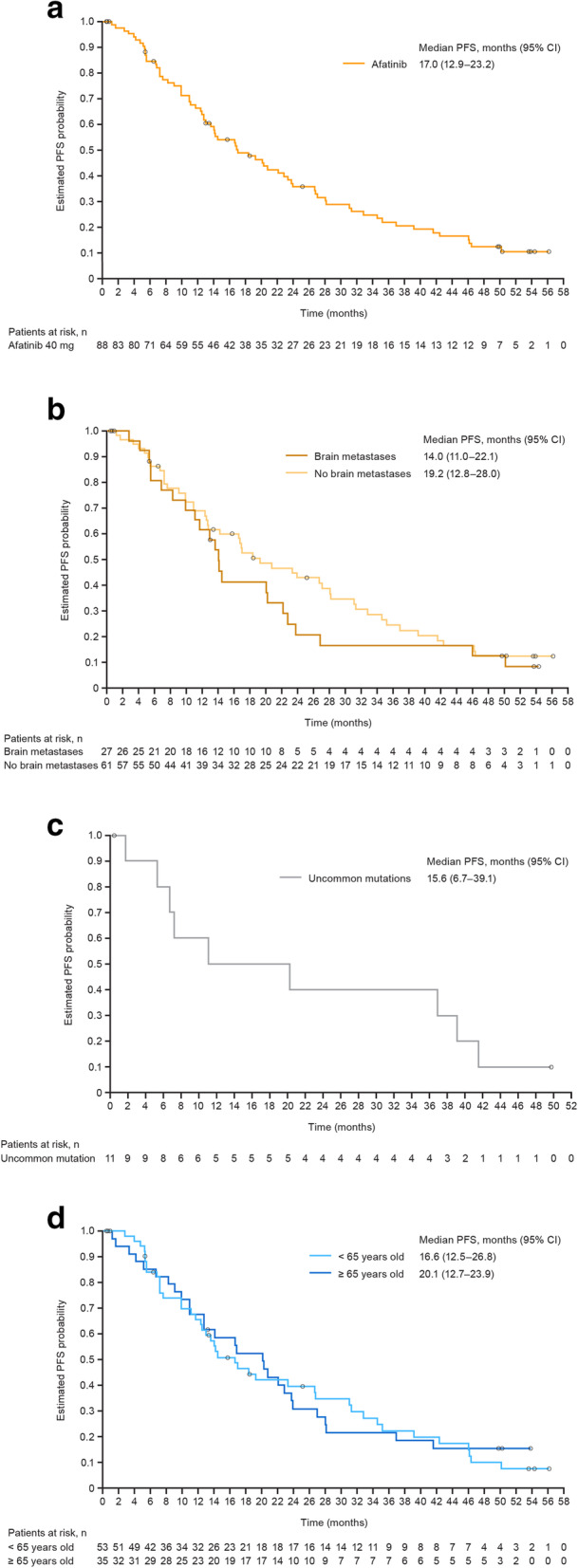
Progression-free survival in (**a**) all treated patients. (**b**) Patients with or without brain metastases at baseline. (**c**) Patients with uncommon *EGFR* mutations (excluding T790M or exon 20 insertions). (**d**) Patients aged < 65 or ≥ 65 years. (CI: confidence interval; PFS: progression-free survival)

Treatment outcomes are summarized in Table 4. Overall median PFS was 17.0 months (95% CI 12.9–23.3 months; Fig. 2A). Median PFS was 20.2 months (95% CI 14.2–27.0 months) in patients who received first-line afatinib (*n* = 66). The ORR was 81%; median duration of response (DOR) was 15.1 months (95% CI 12.4–21.4 months). Fifty-nine (67%) patients had responded within 8 weeks.

**Table 4:** Summary of treatment outcome

Parameter	Treatment Set *N* = 88
PFS	
Patients who progressed or died, *n* (%)	70 (80)
Median, months [95% CI]	17.0 [12.9–23.3]
Tumor response, *n* (%)	
Best overall response	
Complete response	2 (2)
Partial response	69 (78)
Stable disease	12 (14)
Progressive disease	2 (2)
Not evaluable	3 (3)
ORR, *n* (%) [95% CI]	71 (80.7) [70.9–88.3]
Disease control	
DCR, *n* (%) [95% CI]	83 (94.3) [87.2–98.1]
Duration of disease control, months [95% CI]	18.3 [13.6–23.7]

*CI* confidence interval, *DCR* disease control rate, *ORR* objective response rate, *PFS* progression-free survival

Post hoc analysis of PFS in patient subgroups is summarized in Table 5. In patients with brain metastases (*n* = 27), median PFS was 14.0 months (95% CI 11.0–22.1 months; Fig. 2B). In patients with brain metastases and common *EGFR* mutations (*n* = 24), median PFS was 14.1 months (95% CI 11.7–22.8 months). The ORR in patients with brain metastases was 89%. All responses occurred within 8 weeks. The median DOR was 12.2 months (95% CI 9.2–20.3 months). In patients with uncommon *EGFR* mutations (any uncommon mutation: *n* = 16; exon 20 insertions: *n* = 3; T790M: *n* = 2; L861Q: *n* = 6; G719X: *n* = 4; other: *n* = 2), median PFS was 9.0 months (95% CI 5.5–20.2 months). Median PFS in patients with G719X or L861Q mutations was 15.6 months (95% CI 6.7–39.1 months; Fig. 2C). In these patients, the ORR was 55% and the DOR was 17.2 months (95% CI 9.2–35.1 months). Patients who were ≥ 65 years old had similar PFS to those aged < 65 years old (Fig. 2D).

**Table 5:** PFS in patient subgroups

Category	Patient Subgroup	
Age, years	< 65 years	≥ 65 years
N	53	35
Patients who progressed or died, *n* (%)	42 (79)	28 (80)
Median PFS, months (95% CI)	16.6 (12.5–26.8)	20.1 (12.7–23.9)
Brain metastases at screening	Yes	No
N	27	61
Patients who progressed or died, *n* (%)	23 (85)	47 (77)
Median PFS, months (95% CI)	14.0 (11.0–22.1)	19.2 (12.8–28.0)
EGFR mutation type^a^	Common	Uncommon
N	72	16
Patients who progressed or died, *n* (%)	56 (78)	14 (88)
Median PFS months, (95% CI)	19.2 (14.2–23.9)	9.0 (5.5–20.2)
Afatinib line of therapy	First-line	Second-line
N	66	22
Patients who progressed or died, *n* (%)	50 (76)	20 (91)
Median PFS, months (95% CI)	20.2 (14.2–27.0)	12.9 (7.2–26.7)
Patients with an afatinib dose reduction^b^	No	Yes
N	54	34
Patients who progressed or died, *n* (%)	41 (76)	29 (85)
Median PFS, months (95% CI)	20.2 (13.6–31.3)	14.2 (11.7–22.1)

*CI* confidence interval, *EGFR* epidermal growth factor receptor, *PFS* progression-free survival

^a^Common mutations indicates patients whose tumors harbor *EGFR* exon 19 deletions only or L858R substitutions only: uncommon mutations indicates patients whose tumors harbor *EGFR* mutation categories other than exon 19 deletions only and L858R only; ^b^Dose reduction within the first 6 months of treatment

### Cancer-related symptoms

**Fig. 3:**
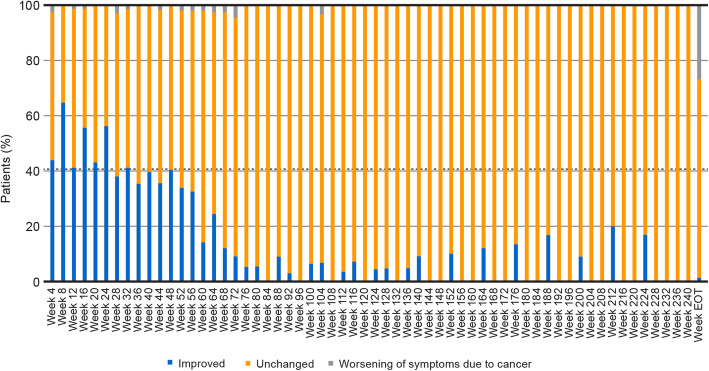
Change in cancer-related symptoms while on treatment according to the investigator. (EOT: end of treatment)

Over the first year of treatment, the maximum percentage of patients deemed to have improved symptoms was 66% (week 8) and the minimum percentage was 34% (week 52; Fig. 3). Patients deemed to have unchanged symptoms ranged from 36% (week 8) to 66% (week 36). A maximum of 3% of patients had worsening of symptoms due to cancer. At the EOT, 1% of patients had improved symptoms, 72% had unchanged symptoms, and 27% had a worsening of symptoms due to cancer.

## Discussion

Real-world data are increasingly recognized by regulatory bodies as an important source of information for the monitoring the safety of approved agents, and to support approvals of agents in development [16]. In this study, patient characteristics were similar to those previously reported in studies of EGFR TKIs used in routine clinical practice in Asia, including Korea [17]. The present study included subsets that are generally under-represented in clinical trials, such as patients with central nervous system (CNS) involvement (31%) and patients with uncommon *EGFR* mutations (18%). In this diverse cohort of patients, the tolerability profile of afatinib was predictable and in line with previous randomized controlled trials [7–9] and real-world studies [16, 18]. However, the rate of TRAEs with afatinib in the current study was higher than observed in randomized controlled trials, possibly reflecting the heterogeneity of patients included the study. While the most common TRAEs were EGFR TKI class-related toxicities (diarrhea, rash/acne and stomatitis) and were not unexpected, the overall rate of grade ≥ 3 TRAEs was 58% compared with 31–49% in the LUX Lung 3, 6 and 7 studies (diarrhea: 5–14%; rash/acne: 9–16%; stomatitis: 4–9%). As expected, given the existence of multiple tablet strengths and previous clinical experience, dose reductions due to AEs were common (56%). Previous analyses of clinical trials [12, 13], and real-world observations [19] have demonstrated that dose reductions of afatinib facilitate the management of TRAEs without significantly affecting activity. The rate of permanent treatment discontinuation due to TRAEs in the current study was low (5%) and was comparable to other studies performed in routine clinical practice (≤ 5%) and randomized clinical trials, (6–8%), suggesting that the use of tolerability-guided dose adjustment in real-world clinical practice can help mitigate common TRAEs [7–9, 16].

In the current study, afatinib stabilized or improved on-treatment cancer-related symptoms in the majority patients. These observations are consistent with clinical trials. For example, in LUX-Lung 3 and 6, afatinib improved global health status and quality of life (QoL) versus chemotherapy and conferred better control of certain key symptoms [20, 21]. Of note, nearly three quarters of patients treated with afatinib had stable, or improved symptoms at the EOT, suggesting that the majority of patients would be sufficiently fit enough to undertake subsequent treatment. This assertion is supported by recent pooled analysis of LUX-Lung 3, 6, and 7 which showed that approximately 70% of patients treated with first-line afatinib subsequently received further systemic therapy [13]. Uptake of subsequent therapy was around 90% in countries with comprehensive reimbursement policies.

In this study, afatinib demonstrated encouraging activity in Korean patients with a median PFS of 17.0 months and an ORR of 81%. Sixty-seven percent of patients responded within 8 weeks. These findings generally compare favorably with afatinib activity reported in Asian patients treated in routine clinical practice (median PFS 12.2–19.1 months) [16] and in LUX-Lung 6 (median PFS 11.0 months, ORR 67%) [8]. Afatinib was observed in patient subgroups, including those with CNS metastases, rare *EGFR* mutations, and the elderly (≥ 65 years). In patients with uncommon mutations, median PFS was 9.0 months (15.6 months when patients with T790M or exon 20 insertions were excluded). These data are in line with other studies. Preclinical data indicate that afatinib has a broad inhibitory profile against a wide range of *EGFR* mutations, including compound mutations [22]. Indeed, some categories of uncommon mutations, such as exon 18 mutations, appear to be more sensitive to second-generation EGFR TKIs than first- or third-generation EGFR TKIs in vitro, suggesting that afatinib could be a preferred treatment option in this setting [23]. In a retrospective analysis of LUX-Lung 2, 3, and 6, median PFS with afatinib in patients with rare *EGFR* exon 18–21 mutations was 10.7 months [24]. Based on these data, afatinib’s label was broadened to include tumors with uncommon activating EGFR mutations, including G719X, L861Q, and S768I. Activity of afatinib against these mutations has also been demonstrated in other ‘real-world’ Asian studies [16]. Osimertinib has also demonstrated activity against uncommon activating EGFR mutations in a single-arm phase II study [25]. Treatment options for exon 20 mutations remain an unmet need, but several agents are in clinical development such as poziotinib and mobocertinib which has shown promise in phase II trials [26, 27].

Recent preclinical data indicate that the CNS penetration of different EGFR TKIs varies and the blood-brain-barrier is more permeable to osimertinib than first- and second-generation EGFR TKIs [28]. Accordingly, sub-analysis of the phase III FLAURA trial demonstrated greater CNS activity with osimertinib than erlotinib or gefitinib [29]. Although no head-to-head data versus osimertinib are available, clinical studies have shown that afatinib is also active in patients with EGFR mutation-positive NSCLC and CNS metastases [16, 30]. The current study also demonstrated encouraging activity of afatinib (median PFS 14.0 months, ORR 89%) in patients with CNS metastases (*n* = 27). Preclinical studies have demonstrated that afatinib can cross the blood brain barrier so that pharmacologically relevant concentrations reach brain lesions. Indeed, activity against CNS lesions has been demonstrated in patients [31, 32]. Also, data indicate that afatinib may be able to protect against CNS progression. Competing risk analysis of LUX-Lung 3 and 6 showed that 24-month risk of de novo CNS progression was 5% and risk of non-CNS progression was 71% [31].

This study has a number of weaknesses. As it was a single-arm, exploratory study with a small sample size, data should be interpreted with caution. Also, mutational analysis of tumors at the point of acquired resistance was not mandated in this study and post-progression therapy was not documented. However, data from other studies indicate that the T790M resistance mutation emerges in around 50–70% of afatinib-treated tumors [33–35], with particularly high detection rates if sensitive plasma-based assays are used [36]. Given that osimertinib is highly active against T790M-positive tumors [37], a sequential regimen of afatinib followed by osimertinib could be considered in many patients with *EGFR* mutation-positive NSCLC, which may delay the requirement for chemotherapy. In a recent global observational study of 203 patients, this sequence conferred median OS of 41.3 months (90% CI 36.8–46.3 months) and median time to treatment failure of 28.1 months (90% CI 26.8–30.3 months) [38]. Further data are required regarding outcomes with afatinib, or dacomitinib, followed by osimertinib, both in clinical trial and real-world settings. In this regard, data from the ongoing phase 2 trials, Heat on Beat (afatinib versus osimertinib [39]), CAPLAND (NCT04811001; dacomitinib/osimertinib versus osimertinib/dacomitinib) and NCT03810807 (dacomitinib followed by osimertinib/chemotherapy versus osimertinib followed by dacomitinib/chemotherapy) are eagerly anticipated. Of note, a recent phase 2 trial of 12 patients indicated that dacomitinib has limited activity on patients who progress on first-line osimertinib (ORR: 17%; median PFS: 1.8 months) [40]. Ultimately, consideration of sequential EGFR TKI regimens will require routine testing for T790M at the point of acquired resistance. Recent real-world evidence in the USA suggests that T790M testing rates may be low (< 20%) in an everyday clinical practice setting [41], although this will vary from country to country. Testing rates may be improved in the future with implementation of liquid biopsy methodologies [42].

In conclusion, afatinib administered to Korean patients treated in routine clinical practice was well tolerated with no unexpected safety signals. TRAEs were detected early, usually within the first few weeks of treatment, predictable, and manageable. Active management of AEs facilitated the encouraging clinical activity of afatinib observed in this study. Afatinib was active in the overall cohort and in selected patient subgroups including those with uncommon *EGFR* mutations or asymptomatic brain metastases at baseline.

## Supplementary Information

**Additional file 1: **Supporting methods. **Table S1.** EGFR mutations in the treated set. **Table S2.** Treatment-emergent adverse events.

## Data Availability

The datasets used and/or analyzed during the current study are available from the corresponding author on reasonable request. If necessary, documents will be redacted and de-identified to protect confidentiality of study participants and personnel. Further details are available at https://trials.boehringer-ingelheim.com/data_sharing/sharing.html#accordion-1-2. Researchers should use https://vivli.org/ to request access to raw data from this study.

## Abbreviations

AE: Adverse event
CNS: Central nervous system
CTCAE: Common Terminology Criteria for Adverse Events
CI: Confidence interval
DOR: Duration of response
ECOG PS: Eastern Cooperative Oncology Group performance status
EOT: End-of-treatment
EGFR: Epidermal growth factor receptor
ILD: Interstitial lung disease
MedDRA: Medical Dictionary for Regulatory Activities
NSCLC: Non-small cell lung cancer
ORR: Overall response rate
OS: Overall survival
PFS: Progression-free survival
QoL: Quality of life
SAEs: Serious adverse events
TEAEs: Treatment-emergent adverse events
TRAEs: Treatment-related AEs
TKIs: Tyrosine kinase inhibitors
