# Supplementary material for: An open-label expanded access program of afatinib in EGFR tyrosine kinase inhibitor-naïve patients with locally advanced or metastatic non-small cell lung cancer harboring EGFR mutations
Source: BMC Cancer. 2021 Jul 12;21:802. doi: 10.1186/s12885-021-08445-9 (PMC8274031; doi:10.1186/s12885-021-08445-9)
Supplement: Supplementary file 1 — Additional file 1: Supporting methods. Table S1. EGFR mutations in the treated set. Table S2. Treatment-emergent adverse events. [file 12885_2021_8445_MOESM1_ESM.docx]

ORIGINAL RESEARCH: surgical oncology, cancer imaging and interventional therapeutics

**An open-label expanded access program of afatinib in EGFR tyrosine kinase inhibitor-naïve patients with locally advanced or metastatic non-small cell lung cancer harboring EGFR mutations**

Keunchil Park^1^, Jin-Soo Kim^2^, Joo-Hang Kim^3^, Young-Chul Kim^4^, Hoon-Gu Kim^5^, Eun Kyung Cho^6^, Jong-Youl Jin^7^, Miyoung Kim^8^, Angela Märten^9^ and Jin-Hyoung Kang^10^

^1^Division of Hematology-Oncology, Samsung Medical Center, Sungkyunkwan University School of Medicine, Seoul, South Korea. ^2^Department of Internal Medicine, Seoul Metropolitan Government Seoul National University Boramae Medical Center, Seoul, South Korea. ^3^CHA Bundang Medical Center, CHA University, Gyeonggi-do, Seongnam, South Korea. ^4^Chonnam National University Medical School, CNU Hwasun Hospital, Gwangju, South Korea. ^5^Department of Internal Medicine, Gyeongsang National University College of Medicine and Gyeongsang National University Changwon Hospital, Changwon, South Korea. ^6^Gil Medical Center, Gachon University College of Medicine, Incheon, South Korea. ^7^Bucheon St Mary’s Hospital, The Catholic University of Korea, Bucheon, South Korea. ^8^Boehringer Ingelheim Korea Ltd, Seoul, South Korea. ^9^Boehringer Ingelheim International GmbH, Ingelheim am Rhein, Germany. ^10^Department of Internal Medicine, Seoul St. Mary's Hospital, The Catholic University of Korea, Seoul, South Korea.

**Corresponding author:** Keunchil Park

Division of Hematology-Oncology, Department of Medicine Samsung Medical Center, Sungkyunkwan University School of Medicine

81 Irwon-ro, Gangnam-gu, 06351 Seoul, South Korea

Email: [kpark@skku.edu](mailto:kpark@skku.edu)

**Supporting methods**

**Treatment interruptions and dose reductions**

Treatment interruptions and dose reductions were permitted for the management of treatment-related adverse events (TRAEs), including any TRAE grade ≥ 3, grade 2 diarrhea persisting ≥ 48 hours, reduced renal function grade ≥ 2, as measured by serum creatinine, proteinuria, or > 50% reduction in glomerular filtration rate versus baseline and drug-related onset or worsening of pulmonary systems (excluding interstitial lung disease). During treatment interruptions, afatinib was suspended until severity recovered to grade ≤ 1, or to baseline severity. If recovery was achieved within 6 weeks, afatinib was resumed at a lower dose by 10 mg decrements down to a minimum of 20 mg/day; otherwise dosing was permanently discontinued. Following a dose reduction, the dose could not be increased later. If 20 mg/day was intolerable, permanent discontinuation was considered.

**Assessments**

Disease assessment was based on the assessment of cancer-related symptoms and, if available, radiologic assessments as per standard of care at the participating site. The investigator was not obligated to perform any radiologic scans as part of this trial. However, if such scans were performed as part of standard of care, this information was to be recorded on the disease assessment case report form.

**Endpoint definitions**

Treatment emergent adverse events were adverse events occurring between first drug administration and 28 days after last treatment administration. For TRAEs, the relationship was defined by the investigator.

Progression-free survival was defined as the number of days from the date of the first administration of afatinib to the date of progression or death (due to any cause), whichever occurred first.

Tumor response was assigned to one of the following categories by the treating investigator according to local standard pattern of care for non-small cell lung cancer (NSCLC): complete response, partial response, stable disease, progressive disease, and not evaluable.

Objective response was defined as a tumor assessment of either complete or partial response. Time to objective response was defined as the number of days from the start of treatment to the first recorded objective response. Duration of objective response was defined as the number of months from first objective response to the time of progression or death (due to any cause).

Best overall response was defined as the best individual tumor response taken from the date of the first administration of afatinib until the earliest recording of progressive disease, death (due to any cause), or end of study treatment.

Cancer-related symptoms were recorded in the electronic case report form (eCRF) based on investigator judgement as either improved, unchanged, or ‘worsening of symptoms due to cancer’. The proportion of patients in each category was calculated in 4-week periods and at the end-of-treatment (EOT) visit.

The overall assessment of clinical benefit was recorded by the investigator in the eCRF. The proportion of patients in each category (‘yes’ or ‘no’) was tabulated in 4-week periods and at the EOT visit.

**Table S1** EGFR mutations in the treated set

| EGFR Mutation Category, *n* (%) | Treatment Set  *N* = 88 |
| --- | --- |
| Exon 19 deletions | 50 (57) |
| Exon 19 deletions/T790M | 1 (1) |
| Exon 20 insertions | 3 (3) |
| G719S, G719A, G719C | 3 (3) |
| G719S, G719A, G719C/T790M | 1 (1) |
| L858R | 22 (25) |
| L858R/L861Q | 5 (6) |
| L861Q | 1 (1) |
| Other | 2 (2) |

*EGFR* epidermal growth factor receptor

**Table S2** Treatment-emergent adverse events

| TEAE Category | Grade 3 | Grade 4 | Grade 5 | All Grades |
| --- | --- | --- | --- | --- |
| Any | 55 (63) | 2 (2) | 7 (8) | 88 (100) |
| Diarrhea | 21 (24) | 0 | 0 | 86 (98) |
| Rash/acne^a^ | 19 (22) | 0 | 0 | 81 (92) |
| Stomatitis^a^ | 13 (15) | 0 | 0 | 69 (78) |
| Pruritus | 3 (3) | 0 | 0 | 41 (47) |
| Paronychia^a^ | 8 (9) | 0 | 0 | 37 (42) |
| Decreased appetite | 7 (8) | 0 | 0 | 30 (34) |
| Nail disorder | 3 (3) | 0 | 0 | 30 (34) |
| Nausea | 5 (6) | 0 | 0 | 21 (24) |
| Dry skin | 0 | 0 | 0 | 18 (20) |
| Cough | 1 (1) | 0 | 0 | 16 (18) |
| Back pain | 0 | 0 | 0 | 14 (16) |
| Dyspnea | 2 (2) | 0 | 1 (1) | 14 (16) |
| Abdominal pain upper | 0 | 0 | 0 | 13 (15) |
| Headache | 2 (2) | 0 | 0 | 13 (15) |
| Productive cough | 0 | 0 | 0 | 10 (11) |
| Vomiting | 2 (2) | 0 | 0 | 10 (11) |
| Dizziness | 1 (1) | 0 | 0 | 9 (10) |
| Fatigue | 3 (3) | 0 | 0 | 9 (10) |
| Nasal inflammation | 0 | 0 | 0 | 9 (10) |
| Asthenia | 2 (2) | 0 | 0 | 8 (9) |
| Palmar-plantar erythrodysesthesia syndrome | 1 (1) | 0 | 0 | 8 (9) |
| Abdominal pain | 5 (6) | 0 | 0 | 7 (8) |
| Insomnia | 1 (1) | 0 | 0 | 7 (8) |
| Oropharyngeal pain | 1 (1) | 0 | 0 | 7 (8) |
| Pleural effusion | 3 (3) | 0 | 0 | 7 (8) |
| Weight decreased | 1 (1) | 0 | 0 | 7 (8) |
| Pyrexia | 1 (1) | 0 | 0 | 6 (7) |
| Pneumonia | 1 (1) | 0 | 0 | 5 (6) |
| Dysarthria | 1 (1) | 0 | 0 | 4 (5) |
| Pulmonary embolism | 1 (1) | 0 | 0 | 4 (5) |
| Generalized edema | 1 (1) | 0 | 0 | 2 (2) |
| Hemorrhoids | 1 (1) | 0 | 0 | 2 (2) |
| Hyperglycemia | 2 (2) | 0 | 0 | 2 (2) |
| Hyperkeratosis | 1 (1) | 0 | 0 | 2 (2) |
| Neoplasm progression | 0 | 0 | 2 (2) | 2 (2) |
| Respiratory failure | 0 | 0 | 2 (2) | 2 (2) |
| Abdominal distension | 1 (1) | 0 | 0 (0) | 1 (1) |
| Ascites | 1 (1) | 0 | 0 | 1 (1) |
| Balance disorder | 1 (1) | 0 | 0 | 1 (1) |
| Blood creatinine phosphokinase | 1 (1) | 0 | 0 | 1 (1) |
| Bradycardia | 0 | 1 (1) | 0 | 1 (1) |
| Cardiac arrest | 0 | 1 (1) | 0 | 1 (1) |
| Death | 0 | 0 | 1 (1) | 1 (1) |
| Deep vein thrombosis | 1 (1) | 0 | 0 | 1 (1) |
| Gastritis erosive | 1 (1) | 0 | 0 | 1 (1) |
| Hydrocephalus | 0 | 0 | 1 (1) | 1 (1) |
| Hypercalcemia | 0 | 1 (1) | 0 | 1 (1) |
| Hypoacusis | 1 (1) | 0 | 0 | 1 (1) |
| Interstitial lung disease | 1 (1) | 0 | 0 | 1 (1) |
| Lower limb fracture | 1 (1) | 0 | 0 | 1 (1) |
| Metastases to central nervous system | 1 (1) | 0 | 0 | 1 (1) |
| Metastases to meninges | 1 (1) | 0 | 0 | 1 (1) |
| Nail toxicity | 1 (1) | 0 | 0 | 1 (1) |
| Onychoclasis | 1 (1) | 0 | 0 | 1 (1) |
| Pain of skin | 1 (1) | 0 | 0 | 1 (1) |
| Pancreatitis | 1 (1) | 0 | 0 | 1 (1) |
| Pelvic fracture | 1 (1) | 0 | 0 | 1 (1) |
| Peripheral motor neuropathy | 1 (1) | 0 | 0 | 1 (1) |
| Pituitary cancer metastatic | 1 (1) | 0 | 0 | 1 (1) |
| Retinal detachment | 1 (1) | 0 | 0 | 1 (1) |
| Road traffic accident | 1 (1) | 0 | 0 | 1 (1) |
| Scrub typhus | 1 (1) | 0 | 0 | 1 (1) |
| Subdural hematoma | 1 (1) | 0 | 0 | 1 (1) |
| Tumor pain | 1 (1) | 0 | 0 | 1 (1) |
| Vitreous hemorrhage | 1 (1) | 0 | 0 | 1 (1) |
| Weight increased | 1 (1) | 0 | 0 | 1 (1) |

*CTCAE* Common Terminology Criteria for Adverse Events, *MedDRA* Medical Dictionary for Regulatory Activities, *TEAE* treatment-emergent adverse event

Shown are *n* (%) TEAEs in > 10% of patients at any grade, all grade 3, 4, and 5 in the patient treatment set. TEAEs are shown by MedDRA version 21.1 preferred terms and highest grade according to CTCAE version 3. ^a^Category of specific grouped preferred terms
